# Microsurgical resection of gliomas of the cingulate gyrus: a systematic review and meta-analysis

**DOI:** 10.1007/s10143-023-02127-9

**Published:** 2023-09-01

**Authors:** Simon Diaz, Nicolas Reyns, Koray Özduman, Marc Levivier, Michael Schulder, Constantin Tuleasca

**Affiliations:** 1 Neurosurgery Service and Gamma Knife Center, Lausanne University Hospital (CHUV), Rue du Bugnon 44-46, BH-08, CH-1011 Lausanne, Switzerland; 2 Neurosurgery and Neurooncology Service, Centre Hospitalier Regional Universitaire de Lille, Roger Salengro Hospital, Lille, France; 3 Department of Neurosurgery, School of Medicine, Neurosurgery Acıbadem Mehmet Ali Aydınlar University, Istanbul, Turkey; 4 Faculty of Biology and Medicine (FBM), University of Lausanne (UNIL), Lausanne, Switzerland; 5 Department of Neurosurgery, Zucker School of Medicine at Hofstra/Northwell, Manhasset, NY USA; 6 Ecole Polytechnique Fédérale de Lausanne (EPFL, LTS-5), Lausanne, Switzerland

**Keywords:** Cingulate gyrus glioma, Microsurgical resection, Mortality, Morbidity

## Abstract

Cingulate gyrus gliomas are rare among adult, hemispheric diffuse gliomas. Surgical reports are scarce. We performed a systematic review of the literature and meta-analysis, with the aim of focusing on the extent of resection (EOR), WHO grade, and morbidity and mortality, after microsurgical resection of gliomas of the cingulate gyrus. Using Preferred Reporting Items for Systematic Reviews and Meta-Analyses guidelines, we reviewed articles published between January 1996 and December 2022 and referenced in PubMed or Embase. Inclusion criteria were peer-reviewed clinical studies of microsurgical series reporting resection of gliomas of the cingulate gyrus. Primary outcome was EOR, classified as gross total (GTR) versus subtotal (STR) resection. Five studies reporting 295 patients were included. Overall GTR was 79.4% (range 64.1–94.7; *I*^2^= 88.13; *p* heterogeneity and *p* < 0.001), while STR was done in 20.6% (range 5.3–35.9; *I*^2^= 88.13; *p* heterogeneity < 0.001 and *p*= 0.008). The most common WHO grade was II, with an overall rate of 42.7% (24–61.5; *I*^2^= 90.9; *p* heterogeneity, *p*< 0.001). Postoperative SMA syndrome was seen in 18.6% of patients (10.4–26.8; *I*2= 70.8; *p* heterogeneity= 0.008, *p*< 0.001), postoperative motor deficit in 11% (3.9–18; *I*^2^= 18; *p* heterogeneity= 0.003, *p*= 0.002). This review found that while a GTR was achieved in a high number of patients with a cingulate glioma, nearly half of such patients have a postoperative deficit. This finding calls for a cautious approach in recommending and doing surgery for patients with cingulate gliomas and for consideration of new surgical and management approaches.

## Introduction

Gliomas are the most common malignant tumors of the central nervous system [1]. Primary involvement of the cingulate gyrus is, on the contrary, a rare theme in adult hemispheric diffuse gliomas, being observed in as low as 3.5% of cases [2]. Secondary involvement of the cingulate gyrus is much more common, especially in glioblastomas, but impingement on the corpus callosum with bilateral extension frequently precludes surgical resection, and therefore, such patients are underrepresented in surgical series [3].

There are few reports about surgical treatment of gliomas in the cingulate gyrus [4], and little is known about the functional outcome in this specific entity of paralimbic tumors.

Studies of the structural anatomy of the cingulate gyrus have recently revealed a four-region neurobiological model, which was proposed based upon structural, circuitry, and functional imaging observations. This model encompasses the anterior cingulate, midcingulate, posterior cingulate, and retrosplenial cortices and explains the multiple neurological deficits that might eventually appear after cingulotomy of various regions [5]. For oncological purposes, the extent of resection (EOR) plays a role in progression-free survival and overall survival. The surgical goal for patients with gliomas remains a maximal safe resection, but what those limits are in patients with cingulate tumors remains uncertain.

Here, we performed a systematic review and meta-analysis of the microsurgical series discussing resection of gliomas of the cingulate gyrus. We focused on several relevant outcomes, including the EOR, WHO tumor grades, and postoperative morbidity and mortality.

## Materials and methods

A PubMed and Embase search was performed for entries between January 1996 and December 2022 using the following MESH terms: (glioma) AND (cingulate) OR (cingulate gyrus) OR (cingulate cortex) OR (cingulum). Inclusion criteria were as follows: peer-reviewed clinical studies of microsurgical series reporting resection of gliomas of the cingulate gyrus, written in English. Exclusion criteria were case reports [6], abstracts, book chapters, and conference papers.

Two independent reviewers (SD, CT) assessed the data by applying the inclusion and exclusion criteria. There were no disagreements.

This study was performed in agreement with the published Preferred Reporting Items for Systematic Review and Meta-Analyses guidelines [7].

**Fig. 1:**
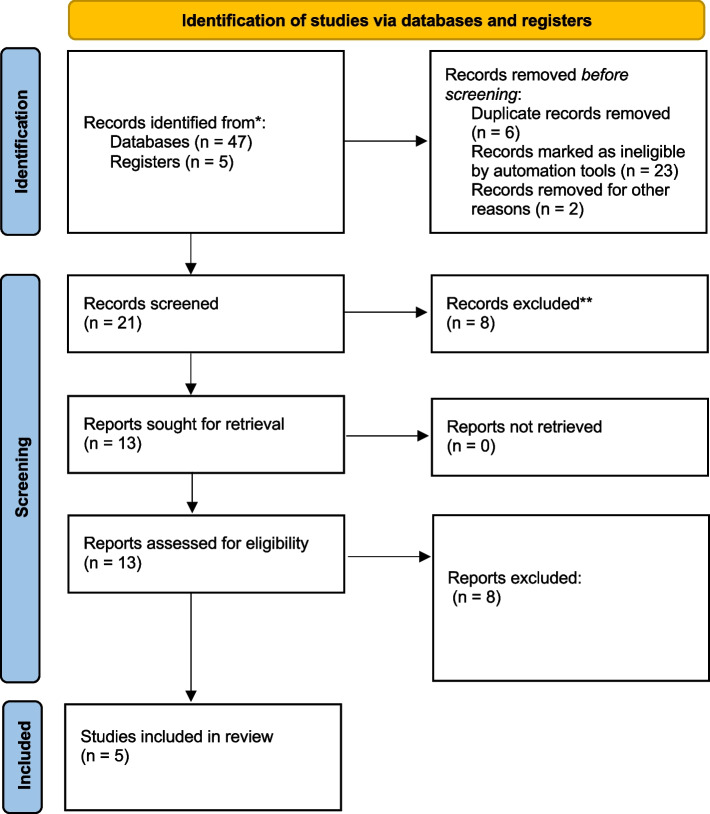
PRISMA flowchart. PRISMA 2020 flow diagram for new systematic reviews which included searches of databases and register only. *Consider, if feasible to do so, reporting the number of records identified from each database or register searched (rather than the total number across all databases/registers). **If automation  tool were used, indicate how many records were excluded by a human and how many were excluded by automation tools

Data extraction was done as per individual studies. We finally report 5 series [2, 4, 8–10] reporting a total number of 295 patients (Fig. 1 and Table 1).

**Table 1:** Basic demographic data

	Number	Age	Side	Anatomical location	Follow-up	Symptom	Preoperative diameter/vol
Steiger et al. (2009) [4]	11	Mean 39 (20–65)	L:R= 3:8	All anterior cingulate	WHO II: mean 29 (10–50) WHO III: mean 48 (12–116)	WHO II • Seizure: 2/11 • Attention deficit: 1/11 • Incidental: 1/11 WHO III • Seizure: 5/11 • Headache: 1/11	WHO II: 3.1 cm (3–3.5) WHO III: 3.8 cm (3.5–7)
von Lehe et al. (2009) [2]	34	Mean 42 (12–69)	L:R= -	Posterior (parietal part): 7/34 (18%) Anterior (frontal part): 31/34 (82%) Solely cingulate: 10/34 (26%)	-	• Seizures: 23/34 (61%) • Hemiparesis 8/34 (24%) • Language problems: 4/34 (12%)	-
Tate et al. (2011) [10]	90	21–65	L:R= 37:47 6 bilateral	Anterior: 9/90 Middle: 31/90 Posterior: 6/90 Anterior + middle: 30/90 Middle + posterior: 13/90 Anterior + middle + posterior: 1/90	-	• Seizure: 52/90 • Palsy: 6/90 • Headache: 8/90 • Sensory change: 3/90 • Dizziness: 2/90 • Asymptomatic 9:90	-
Oszvald et al. (2012) [9]	65	22–75	L:R= 29:36	Anterior: 15/65 Anterior plus extension: 41/65 Posterior: 4/65 Posterior plus extension: 5/65	-	• Seizure: 36/65 • Motor deficit: 11/65 • Others: 2/65 • Aphasia: 2/65	-
Gong et al. (2022) [8]	95	19–70	L:R= 54:41	Anterior: 31/95 Anterior + middle: 17/95 Middle: 26/95 Posterior: 12/95 Posterior + middle: 8/95 Anterior + middle + posterior: 1/95	-	• Seizure: 54/95 • Motor deficit: 20/95 • Headache: 32/95 • Memory deficits: 14/95	-

The research question in PICO format was patients with gliomas of the cingulate gyrus (independently of the WHO grade), who underwent microsurgical resection, with no comparison to other populations, while assessing the EOR and morbidity and mortality after surgery.

### Primary and secondary outcomes

Primary outcome was the EOR. Particular attention was paid to gross total resection (GTR) and subtotal resection (STR). GTR was defined as surgery that removed at least 90% of the tumor visible on MRI, with STR removing less than 90%. We also detailed the WHO grades of tumors, such as described in each study, taking into account the WHO classifications used at that respective time.

Secondary outcome was morbidity and mortality due to surgery.

### Statistical analysis

For our meta-analysis, only studies reporting individual data were analyzed. Because of high variations in study characteristics, a statistical analysis using a binary random-effects model (DerSimonian–Laird method) was performed using OpenMeta[analyst] software (Agency for Healthcare Research and Quality). Weighted summary rates were determined using meta-analytical models. Heterogeneity was tested for each meta-analysis; pooled estimates were obtained for all outcomes. Results of series concerning GTR, STR, and morbidity were compared using a meta-regression with a random effect. *p* values < 0.05 were considered statistically significant.

Using R package (version 2023.06), we assessed the heterogeneity of studies under consideration, particularly for GTR, the primary outcome, and further for STR.

## Results

### Extent of resection: gross total resection

**Fig. 2:**
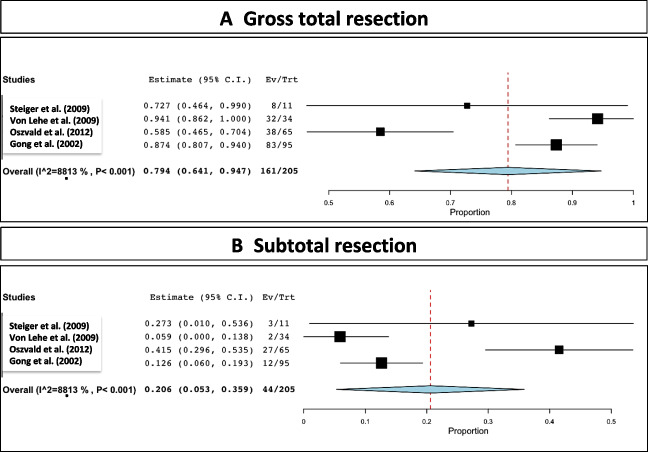
Resection rates. **A** GTR. **B** STR

**Fig. 3:**
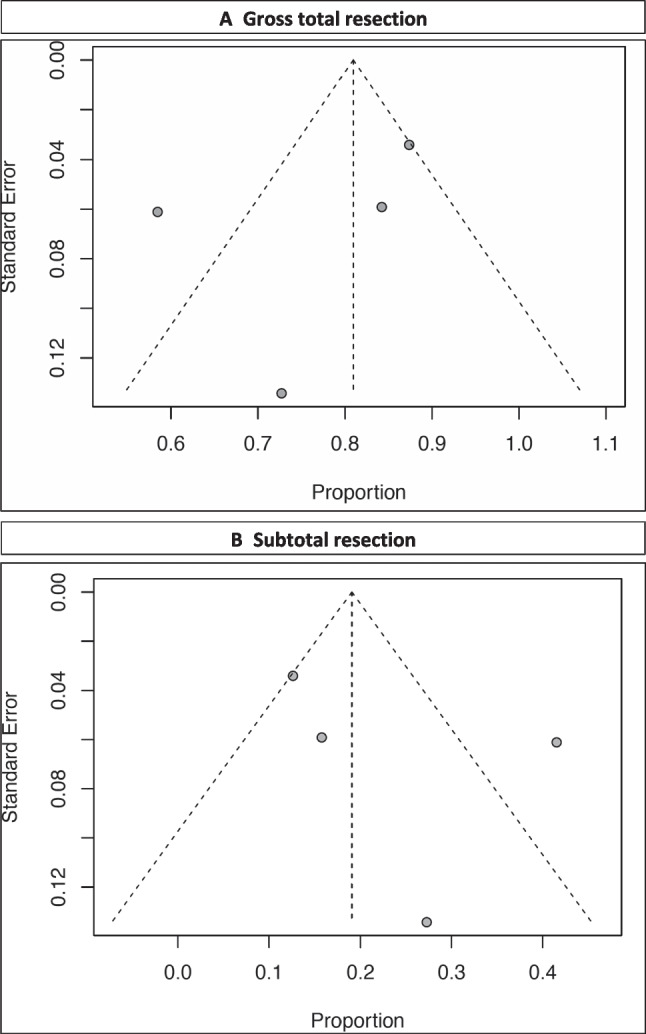
**A** Funnel plot showing the heterogeneity among studies for GTR and **B** funnel plot showing the heterogeneity among studies for STR

Tumor GTR was achieved in 161 out of the 205 reported patients, which corresponded to a rate of 79.4% (range 64.1–94.7; *I*^2^= 88.13; *p* heterogeneity and *p*< 0.001; Fig. 2 A and Table 2). Figure 3A displays a funnel plot showing the heterogeneity among studies for this primary outcome.

**Table 2:** Microsurgical related data (EOR), WHO grade, and morbidity

	Surgery	Diagnosis	Postoperative status	Progression/recurrences	Further therapy	Complications	Death
Steiger et al. (2009) [4]	WHO II • GTR: 3/4 • STR: 1/4 WHO III • GTR: 5/7 • STR: 2/7 WHO II plus III • GTR: 8/11 • STR: 3/11	WHO II • 4/11 (36.4%) WHO III • 7/11 (63.6%)	WHO III: 1 with seizure during follow-up	WHO II • 2/4 (50%) with reoperation (22 and 30 m) WHO III • 4/7 (57.1%) (5, 15, 32, 32 m)	WHO III: ancillary radiotherapy Recurrence: reoperation and RT-CHT depending on histology	WHO III: 1/11 infarction and meningitis	WHO III: 24, 29, 70 months
von Lehe et al. (2009) [2]	• GTR: 32/34 (84%) • STR: 2/34 (16%) Interhemispheric approach: 11/34 (29%) Transcortical approach: 27/34 (71%)	WHO II • 11/34 (29%) WHO III •9/34 (24%) WHO IV • 10/34 26%)	SMA • 13/34 (34%): SMA syndrome (all tumors within anterior cingulate) New deficit • 5/34 (12%) (mild motor or aphasic symptoms) None • 16/34 (47%)	-	-	Major bleeding: 1/34 (2 days after surgery and was in persistent vegetative state) Aseptic meningitis: 1/34	-
Tate et al. (2011) [10]	-	WHO II • 43/90 (47.7%) WHO III • 9/90 (10%) WHO IV • 10/90 (11.1%)	SMA • 18/90 (20%) Motor deficit • 5/90 (5.5%) Sensory disturbances • 2/90 (2.2%) Memory trouble • 1/90 (1.1%) None • 64/90 (71.1%)	-	-	-	-
Oszvald et al. (2012) [9]	• GTR: 38/65 • STR: 27/65	WHO II • 16/65 (24.6%) WHO III • 17/65 (26.1%) WHO IV 32/65 (49.2%)	SMA • 9/65 (13.8%) Motor deficit • 5/65 (7.7%) Other • 9/65 (13.8%) None • 42/65 (64.6%)	• 6/65 (9.2%)	-	-	-
Gong et al. (2022) [8]	• GTR: 83/95 (87.4%) • STR: 12/95 (12.6%)	WHO II • 66/95 (69.5%) WHO III • 18/95 (18.9%) WHO IV • 11/95 (11.6%)	SMA • 21/95 (22.1%) Motor deficit •23/95 (24.2%) Memory trouble • 13/95 (13.7%) Language • 30/95 (31.6%) Epilepsy • 7/95 (7.4%)	-	-	-	-

### Extent of resection: subtotal resection

Tumor STR was achieved in 44 out of the 205 reported patients, which corresponded to a rate of 20.6% (range 5.3–35.9; *I*^2^= 88.13; *p* heterogeneity < 0.001 and *p*= 0.008; Fig. 2B and Table 2). Figure 3B displays a funnel plot showing the heterogeneity among studies for the STR.

### Tumor WHO grade

**Fig. 4:**
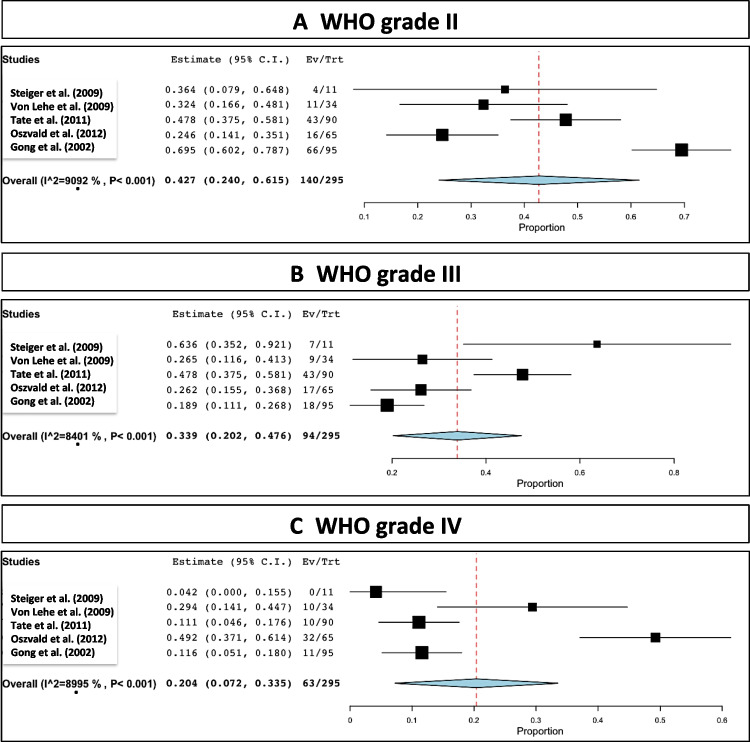
Distribution of WHO grades. **A** II. **B** III. **C** IV

Tumor WHO grade II was found in 140 out of the 295 reported patients, which corresponded to a rate of 42.7% (range 24–61.5; *I*^2^= 90.9; *p* heterogeneity and *p*< 0.001; Fig. 4A and Table 2).

Tumor WHO grade III was seen in 94 out of the 295 reported patients, which corresponded to a rate of 33.9% (range 20.2–47.6; *I*^2^= 84; *p* heterogeneity and *p*< 0.001; Fig. 4B and Table 2).

Tumor WHO grade IV was found in 63 out of the 295 reported patients, which corresponded to a rate of 20.4% (range 7.2–33.5; *I*^2^= 89.9; *p* heterogeneity < 0.001 and *p*= 0.002; Fig. 4C and Table 2).

### Morbidity

**Fig. 5:**
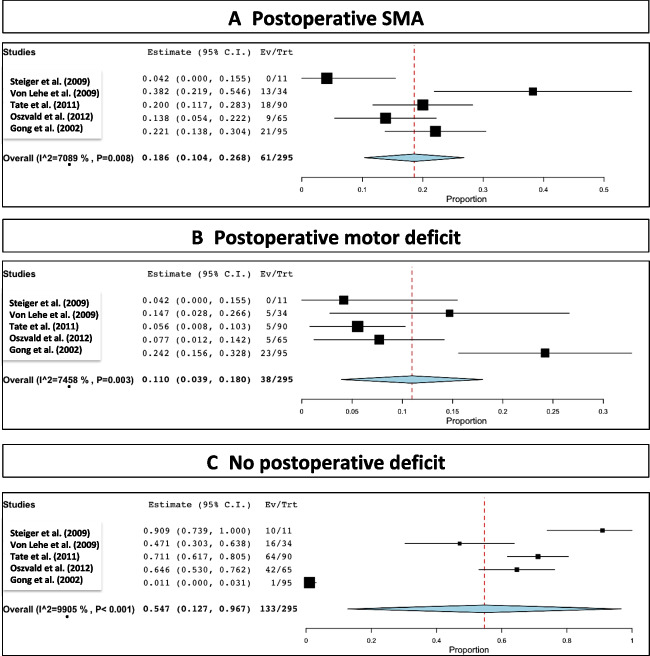
Postoperative status

Postoperative SMA syndrome developed in 61 out of the 295 reported patients, which corresponded to a rate of 18.6% (range 10.4–26.8; *I*^2^= 70.8; *p* heterogeneity= 0.008 and *p*< 0.001; Fig. 5A and Table 2).

Postoperative motor deficit was seen in 38 out of the 295 reported patients, which corresponded to a rate of 11% (range 3.9–18; *I*^2^= 18; *p* heterogeneity= 0.003 and *p*= 0.002; Fig. 5B and Table 2).

No postoperative deficit occurred in 133 out of the 295 reported patients, which corresponded to a rate of 54.7% (range 12.7–96.7; *I*^2^= 99; *p* heterogeneity< 0.001 and *p*= 0.1; Fig. 5C and Table 2).

## Discussion

In the present systematic review and meta-analysis, we reviewed 5 series reporting a total number of 295 patients. Overall tumor GTR was 79.4%, while STR was 20.6%. The most common WHO grade was II (42.7%), followed by III (33.9%) and IV (20.4%), respectively. The overall rate of postoperative SMA syndrome was 18.6% and of motor deficit was 11%. No postoperative deficit was encountered in 54.7%.

With regard to GTR, the overall mean was 79.4%, ranging between 64 and 94%. Such variations are explained by the different involved anatomical regions of the cingulate gyrus, as well as the extensions of gliomas in adjacent eloquent areas. Although neuronavigation, 5-ALA (in selected cases), and electrophysiology are important adjuncts in modern neurooncology, functional sparing by maximal safe resection is a core principle [11]. The anatomical cingulum segregation further explains the heterogenous symptoms and signs at initial presentation and also the potential postoperative deficit, including the most commonly reported SMA syndrome, with an overall rate of 18% (range 10–26%).

The cingulate complex is the supracallosal extension of the hippocampal-parahippocampal region, and it belongs to the hippocampocentric group of paralimbic areas. As it is the case with all allocortical tumors, gliomas of the cingulate gyrus are located in the depth of a fissure, namely, the interhemispheric fissure. The boundaries of the cingulate complex are the paraolfactory area anteriorly, the retrosplenial cortex posteriorly, the cingulate sulcus superiorly, and the sulcus of the corpus callosum inferiorly. Adjacent areas are the falcine and hemispheric surface of F1 with the SMA, the paracentral lobule, and the precuneus in the parietal lobe. First portion of the superior longitudinal fasciculus (SLF-I) is also anatomically and functionally closely associated with the cingulate cortex. Last but not least, the cingulate gyrus surrounds one of the largest association tracts in the brain, the cingulum underneath its cortical surface. Despite this dense connectivity and central location, the hippocampocentric portion of the limbic system is relatively spared by gliomas [3, 12, 13].

Together with the insula, the cingulate gyrus is considered “paralimbic” structure that performs multimodal association of limbic functions and works as an interface between the allo- and neo-cortex [12]. A multitude of functions including memory (cingulum as part of the Papez circuit [14–16]), volitional motor control (anterior cingulate cortex as a part of the cinguloopercular control network), social cognition (cingulum as a major associative tract in mentalizing [17]), internally directed cognition (posterior cingulate as a part of the “default mode network”[18]), motivation, emotion, pain perception, and visceral function were attributed to the cingulate complex. This connectivity is causally related to glioma-tumor biology, as demonstrated by the fact that gliomas involving the “hippocampocentric” limbic system (a.k.a. the Papez circuit) have a more aggressive course compared to gliomas of the “olfactocentric” half [13].

Early studies of surgery on the cingulate gyrus discussed stereotactic neurosurgery for intractable pain [19], obsessive compulsive disorders [20], or depression [21]. Surgery for morphologic anomalies including tumors was and remains uncommon. Schwartz was the first scholar to realize a correlation between anatomical localization and behavior of gliomas [22]. In his later book, Schwartz dedicated a special section on the involvement of the cingulate gyrus, defined gliomas of the anterior, middle, and posterior cingulate gyrus, as well as gyrus rectus gliomas involving the parolfactory area. In the same book, Schwartz also documented co-involvement of the cingulate gyrus together with the corpus callosum resulting in bilateral tumor extension. The first surgical series of cingulate gliomas was reported by Yaşargil in the context of “limbic” tumors [23]. Yaşargil in his later book divided cingulate gliomas into anterior, middle, and posterior subtypes, with invasion of the septal and mediodorsal superior frontal gyrus, paracentral/precuneal gyri, and parahippocampal gyrus/precuneus, respectively. Yasargil also highlighted the importance of protection of callosal arterial plexus during cingulate glioma surgery [24]. In their analysis on the morbidity of cingulate glioma surgery, Tate et al. [10] defined another very rare subtype, namely, tumors of the retrocallosal area, and indicated that the surgical trajectory is the main determinant of morbidity in cingulate glioma surgery.

Anatomical particularities of this region explain the pre- and postoperative symptomatology. It has been previously acknowledged that the cingulate cortex divides into four functionally distinct regions: anterior cingulate cortex (ACC), midcingulate cortex, posterior cingulate cortex (PCC), and retrosplenial cortex [25]. This anatomical segregation explains the presenting heterogenous symptoms and signs at discovery, as well as further possible postoperative deficit. The anterior cingulate cortex comprises Brodmann’s areas (BA) 24, 25, 32, and 33 and is mainly responsible for processing emotions and regulating the endocrine and autonomic responses to emotions. The midcingulate cortex is involved in cognitive processing, specifically reward-based decision-making [26]. Moreover, subcortical connection from MCC to M1 or SMA may play a role in movement planning and even speech initiation [27]. The cingulate motor areas process information from our internal and external states (e.g., emotional state signals from the limbic system) and further translate them into motor commands executed by the primary and supplementary motor cortices and spinal cord [28]. The PCC is responsible for visuospatial orientation, while the retrosplenial cortex mediates imagination, formation, and consolidation of episodic memory. Overall, the cingulate gyrus is an important part of the so-called Papez circuit, a fundamental connective network governing emotional function, linking the hippocampal formation, fornix, anterior thalamic nucleus, cingulum, and entorhinal cortex [29].

The most commonly reported clinical manifestation at discovery was seizure. Particularly, anterior cingulate epilepsy has a broad range of clinical manifestations, as related to the multiple projections of the anterior cingulate into motor systems [30]. Such manifestations can include brief motor seizures occurring during sleep, but also absence, hypermotor, and postural tonic seizures [31], with different areas of onset and patterns of spread [30]. Lesional anterior cingulate epilepsy is characterized by early onset, drug resistance, and behavior disturbances [32].

The structural anatomy of the cingulate gyrus and the four-region model explains the anatomical particularities of the cortical infiltration along with the neurological deficits that might appear after microsurgical resection. However, the individual studies included here did not report separately such outcomes based on the individual sub-regions of the cingulum. Thus, it remains very difficult to correlate the outcomes with the specific subdivisions contained in the four-region model. Gliomas arising from the cingulate gyrus are rare, with extensive resection seemingly safe [2], and an overall GTR reported here of 70.2%. In the particular case of gliomas arising from the anterior and middle cingulate gyrus, an SMA syndrome has to be considered, particularly for tumors extending to the supracingular cortex [2]. Some authors perform even a combined subpial/interhemispheric approach in order to reduce the risk of vascular injury and allow a precise anatomo-surgical dissection [33].

Anterior cingulate gliomas exhibited, in some series, distinct features with regard to presenting symptoms, MRI, histopathology, and prognosis [4]. Within a set of frontal gliomas, the anterior cingulate tumors have a more favorable prognosis [4]. The fronto-mesial glioma WHO grades II and III can be topographically divided into tumors arising from the anterior cingulate gyrus, from the genu of the corpus callosum, or from the gyrus rectus. The prognosis of anterior cingulate gliomas appears to be better than with the other frontal locations [34, 35]. One possible explanation for this may include the relatively small tumor volume, the possible limitation of infiltration by adjacent transverse fiber tracts, or the oligodendrocytic predominance [4]. The infiltration of the subcallosal area is considered a common site of recurrence [4], as such zones posteriorly and towards basal ganglia cannot be safely resected. The lower grade of many resected tumors should not be necessarily taken as an indication for radical resection, as any patient with a tumor in an eloquent region is at risk for permanent deficit after surgery [36].

Our present meta-analysis has several inherent limitations. The first is related to the lack of detail regarding the involved anatomical areas and without specific details of the respective GTR, STR, and morbidity associated with resection from each region. The second is the absence of a detailed pre- and postoperative neuropsychological assessment. The third is related to the limited detail about surgical approaches. The fourth is the absence of details of further local control and overall survival.

## Conclusion

This review found that while a GTR was achieved in a high number of patients with a cingulate glioma, nearly half of such patients have a postoperative deficit. This finding calls for a cautious approach in recommending and doing surgery for patients with cingulate gliomas and for consideration of new surgical and management approaches. The physiological complexity of this region, and the complexity of achieving a surgical cure, should cause neurosurgeons to exercise special care in the surgical approach and extent of tumor removal. In addition, imaging review should help to identify those patients whose tumors are in the anterior region of the cingulate cortex, in which surgery can be done with a lower neurological risk.

## Data Availability

Not applicable
